# Protective Actions of PPAR-*γ* Activation in Renal Endothelium

**DOI:** 10.1155/2008/635680

**Published:** 2009-02-26

**Authors:** Peter E. Westerweel, Marianne C. Verhaar

**Affiliations:** ^1^Department of Vascular Medicine, University Medical Center Utrecht, Heidelberglaan 100, 3584 CX Utrecht, The Netherlands; ^2^Department of Internal Medicine, St. Antonius Hospital, Koekoekslaan 1, 3435 CM Nieuwegein, The Netherlands

## Abstract

Renal endothelial damage is pivotal in the initiation and progression of renal disease. Damaged renal endothelium may be regenerated through proliferation of local endothelium and circulation-derived endothelial progenitor cells. Activation of the PPAR-*γ*-receptors present on endothelial cells affects their cellular behavior. Proliferation, apoptosis, migration, and angiogenesis by endothelial cells are modulated, but may involve both stimulation and inhibition depending on the specific circumstances. PPAR-*γ*-receptor activation stimulates the production of nitric oxide, C-type natriuretic peptide, and superoxide dismutase, while endothelin-1 production is inhibited. Together, they augment endothelial function, resulting in blood pressure lowering and direct renoprotective effects. The presentation of adhesion molecules and release of cytokines recruiting inflammatory cells are inhibited by PPAR-*γ*-agonism. Finally, PPAR-*γ*-receptors are also found on endothelial progenitor cells and PPAR-*γ*-agonists stimulate progenitor-mediated endothelial repair. Together, the stimulatory effects of PPAR-*γ*-agonism on endothelium make an important contribution to the beneficial actions of PPAR-*γ*-agonists on renal disease.

## 1. INTRODUCTION

PPAR-*γ*-agonists are widely used for their insulin-sensitizing actions in the treatment of type 2 diabetes mellitus, but have additional therapeutic potential beyond the metabolic effects. Currently, the clinically most used PPAR-*γ*-agonistic drugs are the thiazolidinediones (TZDs). PPAR-*γ*-agonists may favorably affect the course of renal disease in both diabetic and nondiabetic conditions [1, 2]. In nondiabetic animals, beneficial effects have been shown for anti-GBM antibody-induced crescentic glomerulonephritis [3], passive Heymann nephritis [4], the development of glomerulosclerosis after 5/6 nephrectomy [5], renal ischemia-reperfusion induced damage [6], and anti-Thy-1-glomerulonephritis [7]. One potential mechanism is the modulation of endothelial cell function through activation of PPAR-*γ* receptors, which are expressed on glomerular endothelium [8, 9]. In response to injury, the endothelial expression of PPAR-*γ*-receptors may be increased, for example, as transiently occurs after ischemia- reperfusion [8, 9]. Treatment with PPAR-*γ*-agonists also increases the expression of PPAR-*γ*-receptors on renal endothelium in both the glomerulus and the capillary endothelium of the medullary vasa recta [10]. The relevance for renal disease of activating the PPAR-*γ*-receptors on renal endothelium is becoming increasingly clear and is the focus of this review.

## 2. THE ROLE OF ENDOTHELIUM IN RENAL DISEASE

Renal microvascular endothelial injury is a pivotal pathogenic factor for various renal diseases. Renal disease conditions involving prominent endothelial damage include ischemic nephropathy, glomerulonephritis, interstitial nephritis, and allograft rejection [11, 12]. Endothelial dysfunction and attenuated angiogenesis contribute to declining renal function with ageing [13] and the pathogenesis and progression of chronic kidney disease [11]. The microvascular endothelium, by the release of endothelium-derived factors such as nitric oxide (NO) and as a critical component of the glomerular filtration barrier, exerts important protection against progressive renal damage. Endothelial dysfunction results in increased permeability causing passage of macromolecules (microalbuminuria), which is considered to be the earliest renal sign of vascular dysfunction [14, 15]. Endothelial dysfunction has been shown to predict susceptibility to renal damage in a rat renal injury model [16].

Progression of renal disease does not only depend on the degree of microvascular endothelial injury, but also on the effectiveness of endothelial repair. Impaired glomerular capillary repair was found to be associated with the development of glomerulosclerosis and renal failure [17]. During experimental glomerulonephritis, angiogenic factors such as VEGF and bFGF are released, which stimulate endothelial regeneration [18–20]. Blocking the VEGF-induced endothelial repair with a VEGF-antagonist interferes with renal recovery and results in progressive renal impairment [21]. Consistently, progressive renal disease is associated with reduced expression of angiogenic growth factors and enhanced expression of antiangiogenic factors [22, 23]. The glomerular endothelium can recover from injury by replacing lost or damaged endothelial cells, in part through proliferation of local endothelium, stimulated by the release of angiogenic growth factors [11, 12]. We [24–26] and others [27] have observed in both human and experimental animal studies that damaged glomerular endothelium may also be regenerated from circulating bone marrow-derived endothelial progenitor cells. Endothelial progenitor cells incorporate into the damaged glomerulus, differentiate into mature endothelial cells, and eventually fully integrate into the resident endothelium [26].

Enhancing renal endothelial repair offers therapeutic potential. Stimulating angiogenesis with VEGF-treatment augments capillary repair and renal recovery after glomerulonephritis [19]. Enhancing NO production by supplementing the substrate L-arginine improves the clinical course of anti-Thy-1-glomerulonephritis [28]. Infusion of unselected bone marrow cells ameliorates experimental progressive glomerulosclerosis [29]. Intrarenal administration of endothelial progenitor cells attenuates endothelial injury and mesangial activation in experimental glomerulonephritis [30].

## 3. THE ROLE OF PPAR-*γ*-RECEPTORS IN VASCULAR DEVELOPMENT AND REMODELING

Phenotypical studies in humans and animals with genetic mutations in the PPAR-*γ*-receptor imply a role in the regulation of vascular function and remodeling. In humans with dominant-negative heterozygous mutations in the PPAR-*γ*-receptor causing an impaired capacity for transcriptional activation, diabetes and also hypertension occur at an unusually young age [31]. Heterozygous knockout mice have increased insulin sensitivity [32] and decreased fat mass [33], but do not have a vascular phenotype [34]. However, but PPAR-*γ*-null mice are embryonically lethal due to placental dysfunction, characterized by defective trophoblast differentiation and markedly impaired placental vascularization [34]. A surviving PPAR-*γ*-knockout mouse that was supplemented with a wild-type placenta developed an apparently normal vascular system during further embryogenesis, but died some days after birth due to a combination of pathologies, including severe lipodystrophic changes and hemorrhages [34]. In a conditional knockout model using a cre-lox system to save floxed PPAR-*γ*-knockout mice from embryonic lethality by preserving PPAR-*γ*-function in the trophoblast marked lipodystrophy was also observed, together with insulin resistance. Surprisingly, these mice were hypotensive and showed increased endothelium-dependent relaxation in response to acetylcholine [35]. In the latter study, PPAR-*γ*-function was deficient in all cell types. Mice in which PPAR-*γ*-function was selectively knocked out in endothelial cells only using again a cre-lox system were found to be hypertensive when fed a high-fat diet [36]. Also, in mice with a nonlethal-dominant negative mutation in the PPAR-*γ*-receptor, endothelium-dependent blood vessel dilatation is impaired, while the endothelium is more sensitive to endothelin-1-induced vasoconstriction [37]. Superoxide levels in these mice are elevated and treatment with a superoxide scavenger can reverse the impaired endothelial vasodilation, highlighting the pivotal role of increased radical formation [37]. Cerebral arterioles were hypertrophied with a decrease in luminal diameters, indicative of adverse inward vascular remodeling [37]. Of note, dominant negative PPAR-*γ*-mutations were found not to be fully selective, therefore, it cannot be excluded that some of the effects observed in the knockout systems are attributable to PPAR-*γ*-receptor-independent signaling, in particular through the alternative PPAR-receptors [38].

## 4. EFFECTS OF PPAR-*γ*-ACTIVATION ON ENDOTHELIAL PROLIFERATION, MIGRATION, ANGIOGENESIS, AND APOPTOSIS

Reports on the direct effects of PPAR-*γ*-agonist treatment on proliferation, migration, and angiogenic network formation of cultured endothelial cells showed variable results. Fukunaga et al. demonstrated increased proliferation with both troglitazone and pioglitazone in four different endothelial cell lines [39]. In contrast, two other studies found troglitazone to inhibit proliferation of macrovascular endothelial cells [40] and human umbilical vein endothelial cells [41]. Rosiglitazone was shown also to inhibit proliferation in one study [42], while another study found no effect at all [40].

VEGF-induced endothelial migration was found to be inhibited by PPAR-*γ*-agonists troglitazone and ciglitazone, which was mediated by inhibition of Akt [43]. This is in line with the reduced migration and inhibition of angiogenic network formation by PPAR-*γ*-agonism observed by Xin et al [44]. However, in the study by Fukunaga et al., troglitazone stimulated both endothelial cell migration and proliferation, resulting in accelerated coverage of a disrupted endothelial monolayer in a wound healing assay [39]. Also, Biscetti et al. found stimulation of endothelial network formation with PPAR-*γ* activation using the GW1929 compound, mainly through a VEGF-dependent mechanism [45].

In vitro effects of PPAR-*γ*-agonism on endothelial cell apoptosis have been similarly variable. Both spontaneous and TNF-alpha-induced endothelial apoptosis were shown to be inhibited by various thiazolidinedione PPAR-*γ*-agonists [46] and troglitazone was found to markedly reduce apoptosis in serum-starved endothelial cells [47], while another study observed induction of apoptosis using both ciglitazone and PPAR-*γ*-receptor overexpression [48]. In several studies, the endogenous PPAR-*γ* ligand 15-deoxy-delta12,14-prostaglandin J2 (15d-PGJ2)-induced endothelial cell apoptosis [46, 48, 49], but it is important to note that in vascular endothelial cells, the effects of 15d-PGJ2 may be independent of PPAR-*γ*-receptor activation [49, 50].

Taken together, it is clear that PPAR-*γ*-agonists may modulate endothelial proliferation, migration, angiogenesis, and apoptosis in vitro, but that this may result in both stimulation and inhibition. The factors that determine whether treatment with a PPAR-*γ*-agonist results in a stimulatory or inhibitory effect on endothelial cells remain largely unidentified. This may involve PPAR-*γ*-receptor-independent effects as reported for 15d-PGJ2 [49, 50] and also with the various TZDs [45]. Fukunaga et al. posed that a concentration dependency may explain some of the observed discrepancies, as they observed stimulation of DNA-synthesis at low PPAR-*γ*-agonist dosages and inhibition at higher dosages [39]. However, this cannot explain all of the divergent findings.

In vivo studies in diabetic animals with impaired angiogenesis in response to peripheral ischemia showed that treatment with PPAR-*γ*-agonist pioglitazone augmented the angiogenic response and increased blood flow recovery [51]. In rats with experimental focal cerebral ischemia, PPAR-*γ*-agonist treatment stimulated local angiogenesis and improved functional neurological recovery [52]. In contrast, PPAR-*γ*-agonist treatment inhibited pathological choroidial and retinal neovascularization [53] and suppressed tumor growth and metastasis by inhibiting tumor angiogenesis in several primary tumors, in part through decreasing VEGF-production by tumor cells and blocking the production of angiogenic ELR+CXC-chemokines, mediated through antagonizing NF-kappaB activation [54, 55]. These findings suggest that PPAR-*γ*-agonism may differentially affect neovascularization with inhibition of pathological neovascularization and augmentation of physiological neovascularization. This is in line with the in vitro observations of both stimulatory and inhibitory actions.

## 5. EFFECTS OF PPAR-*γ*-ACTIVATION ON ENDOTHELIAL DYSFUNCTION

Endothelial integrity does not only rely on the number of cells, but also on their function. NO-production is a key component of endothelial function. NO stimulates vasodilation, inhibits inflammation, prevents platelet activation, and scavenges radicals [56]. In vitro studies on the effect of PPAR-*γ*-activation have consistently shown a stimulating effect on NO production by endothelial cells [57–60]. The observation that siRNA against PPAR-*γ* blocked the increase in NO-production confirmed that this effect is PPAR-*γ*-receptor mediated [59]. In both experimental animal studies and in humans, PPAR-*γ*-activation has been shown to augment systemic NO-production and endothelial function. In diabetic rats, PPAR-*γ*-agonist treatment restored impaired endothelium-dependent arterial relaxation by increasing NO-production while reducing oxidative stress [61]. In healthy human subjects, a single dose of troglitazone increased NO-dependent endothelial function measured by venous occlusion plethysmography of the forearm and nitrite levels [62]. In nondiabetic patients with hypertension or hypercholesterolemia, endothelial function was improved with pioglitazone [63]. In type 2 diabetic patients, vascular resistance was shown to be reduced with troglitazone treatment [64, 65] and in type 2 diabetic patients with angina pectoris, troglitazone reduced the frequency of angina pectoris with improving endothelial function [66]. Rosiglitazone attenuated the detrimental effects of the presence of diabetes on NO-production measured directly using an intravital probe and by assessing blood flow in the peripheral skin [67]. Long-term treatment with pioglitazone resulted in a reduced pulse wave velocity, indicative of reduced vascular stiffness [68]. In renal transplant recipients, PPAR-*γ*-agonist treatment enhanced endothelial function [69].

Importantly, the observed beneficial effects on systemic endothelial function were found to extend to improving intrarenal NO-production. In human type 2 diabetic patients, rosiglitazone treatment increased intrarenal NO levels, which was associated with improvement of renal hemodynamics and reduction of proteinuria [70]. This is in line with animal studies. In obese Zucker rats, PPAR-*γ*-agonist treatment lowered blood pressure and ameliorated abnormal pressure natriuresis in association with increased renal NO-metabolite nitrite/nitrate production [71]. In obese hypertensive Sprague-Dawley rats, PPAR-*γ*-agonist treatment also reduced blood pressure, increased NO-metabolite nitrite/nitrate excretion, and reduced excretion of oxidative-stress associated urinary isoprostanes and lipid peroxides [72]. In the kidneys of these rats, eNOS expression was increased while the pathological increase in p47phox and gp91phox associated with obesity was attenuated [72].

Differences have been observed between the various PPAR-*γ*-activating compounds in the signaling level at which NO-production is stimulated. The NO producing enzyme endothelial nitric oxide synthase (eNOS) is not only regulated at the level of transcription and translation, but also has multiple phosphorylation sites for activation and deactivation of the enzyme and requires translocation to the caveolae to associate with its cofactors and effectively produce NO [73, 74]. Troglitazone has been shown to enhance NO-production by increasing eNOS transcription and eNOS protein translation in isolated endothelial cells [58], while no effect was found at the transcriptional level for 15d-PGJ2 [57, 58], pioglitazone [58], and ciglitazone [57]. 15d-PGJ2 and rosiglitazone, but not ciglitazone, were found to stimulate eNOS-phophorylation at activation site ser-1177, thought to be mediated through increasing heat shock protein (hsp)-90 association with eNOS [59]. Interestingly, there is a cross-talk between NO and PPAR-*γ* pathways as NO has been shown to rapidly and dose-dependently increase PPAR-*γ*-binding, mediated by p38 MAPK activation [60].

In vivo, the stimulatory effect of PPAR-*γ*-agonists rosiglitazone and pioglitazone on endothelial NO-production was associated with increased eNOS-phosphorylation, while eNOS mRNA and total protein levels were not affected [51, 75]. Rosiglitazone treatment has been shown to enhance NO-production through enhancement of cellular transport of arginine, the substrate for NO [76]. This is particularly relevant for renal disease, as arginine transport was found to be markedly impaired in uremic conditions [77–79].

PPAR-*γ*-agonism favorably affects other endothelium-derived factors that act in conjunction with NO to maintain endothelial homeostasis. The release of C-type natriuretic peptide, another vasodilatory peptide, by endothelial cells is increased by PPAR-*γ*-agonist treatment [39]. In addition, PPAR-*γ*-agonist treatment increased Cu^2+^ and Zn^2+^-superoxide dismutase expression in cultured endothelial cells, thereby increasing their potential for oxygen radical scavenging [80, 81]. Also, PPAR-*γ*-agonist treatment decreases the production of radical oxygen species in endothelial cells [81, 82], in part through decreasing the expression of subunits of NADPH-oxidase [80, 81].

## 6. ANTIHYPERTENSIVE EFFECTS OF PPAR-*γ*-AGONISM MEDIATED BY THE ENDOTHELIUM

Improving NO-availability is thought to be a major mechanism mediating the blood pressure lowering effect of PPAR-*γ*-agonist treatment. Pioglitazone treatment prevented hypertension and renal oxidative stress both by reducing free-radical production and by increasing nitric oxide production [72]. No blood pressure reduction was seen with PPAR-*γ*-agonist treatment in rats also receiving NO-inhibitor L-NAME [83]. However, other mechanisms may play a role in the anti-hypertensive effect of PPAR-*γ*-agonist treatment, such as effects on the contractility and proliferation of smooth muscle cells [84].

A naturally occurring antagonist of NO is the vasoconstrictor endothelin-1, which is involved in atherosclerosis and hypertension [85]. PPAR-*γ*-activation inhibits the production of endothelin-1 from endothelial cells in vitro [39, 86–88]. In type 2 diabetic patients, pioglitazone treatment reduced urinary endothelin-1 secretion, along with decreasing microalbuminuria [89]. Interestingly, in this study, serum endothelin-1 levels were not affected, suggesting a specific pathogenic role for endothelin-1 secretion in the kidney [89]. In DOCA-salt hypertensive rats, TZD treatment reduced endothelin-1 production and blunted radical oxygen species production with diminished hypertension progression and vascular remodeling [90]. Several studies show that the inhibitory effect of PPAR-*γ*-activation on endothelin-1 secretion takes place at the transcriptional level [86, 87] and it was found to be NO-dependent [87]. Delerive et al. demonstrated that PPAR-*γ*-activation negatively interferes with the activator protein-1 signaling cascade, resulting in inhibition of thrombin-induced transcription of endothelin-1 [88].

In hypertension, increased production and secondary effects of angiotensin-II play a major role. In endothelial cells, angiotensin-II is a strong inducer of NADPH-oxidase, resulting in the production of radical oxygen species [91]. Interestingly, there is a cross-talk between the Angiotensin-II and PPAR-*γ* signaling pathways. Infusion of angiotensin-II downregulates PPAR-*γ*-receptor expression in the vascular wall [92] and treatment with PPAR-*γ*-agonists abrogates many of the angiotensin-II pathophysiological effects [93]. Of note, angiotensin-II-receptor-1 antagonists have been shown to act as partial PPAR-*γ*-agonists [94].

## 7. EFFECTS OF PPAR-*γ*-ACTIVATION ON INFLAMMATORY CELL RECRUITMENT TO THE ENDOTHELIUM

Endothelial cells form an important barrier between the blood and peripheral tissues and regulate homing, adhesion, and transmigration of inflammatory cells. Upon activation, endothelial cells may further release inflammatory cytokines and express adhesion molecules to attract inflammatory cells. PPAR-*γ*-agonist treatment inhibits the increased expression of adhesion molecules such as VCAM-1, ICAM-1, and E-selectin and release of inflammatory cytokines upon stimulation of endothelial cells with PMA [95, 96], TNF-*α* [95, 97], IFN-*γ* [98], IL-1*β* [99], LPS [96], microparticles [100], and high glucose [101]. In vitro studies confirmed that this resulted in decreased adhesion of inflammatory cells to the endothelium [96, 97, 101, 102]. In addition, a recent study showed that in activated primary human brain endothelial cells, PPAR-*γ*-activation resulted in a marked reduction of monocyte adhesion and in vitro transendothelial migration, mediated by inhibition of Rac1 and RhoA GTPases [102]. In vivo, troglitazone and 15d-PGJ2 reduced ICAM-1 and VCAM-1 expression on endothelial cells and reduced inflammatory cell homing to atherosclerotic plaques in a mouse model [103].

## 8. EFFECTS OF PPAR-*γ*-ACTIVATION ON ENDOTHELIAL PROGENITOR CELLS

A recently uncovered effect of PPAR-*γ*-agonists is the capacity to augment the level and function of endothelial progenitor cells. PPAR-*γ*-agonist treatment increases endothelial progenitor cell levels in mice [104, 105] and humans [104, 106, 107]. A stimulatory effect on endothelial progenitor cell outgrowth was also observed when cultured peripheral blood mononuclear cells were exposed to PPAR-*γ*-agonist treatment ex vivo [104, 105, 108, 109], suggesting an effect on endothelial progenitor cell survival, adhesion, or differentiation. PPAR-*γ*-agonist treatment largely prevented apoptosis of endothelial progenitor cells induced by CRP [109] and H_2_O_2_ [105]. Based on marker expression patterns, the differentiation of endothelial progenitor cells toward the endothelial lineage appears to be stimulated [104, 108], while expression of smooth muscle cell markers is inhibited [104]. Pioglitazone inhibited detrimental effects of angiotensin-II on endothelial progenitor cells, including inhibiting the induction of cellular senescence of endothelial progenitor cells via downregulation of the expression of angiotensin-II-receptor-1 and limiting the angiotensin-II-induced increased generation of peroxynitrate and superoxide by the NADPH-oxidase subunit gp91phox [110].

Functionally, PPAR-*γ*-agonist treatment was shown to stimulate angiogenic network formation by endothelial progenitor cells in vitro [109] and in vivo [105]. The adhesion capacity may be increased upon TZD-treatment [108, 109]. In addition, endothelial progenitor cell-mediated reendothelialization of a denuded segment of the femoral artery in mice was accelerated in PPAR-*γ*-agonist treated animals [104]. As endothelial progenitor cells may also participate in regeneration of damaged renal endothelium [26], we investigated a potential role for enhanced endothelial progenitor cell homing and glomerular incorporation by PPAR-*γ*-agonist treatment [7]. However, using a rat allogenic bone marrow transplantation model, we could not detect an effect on the number of incorporated circulation-derived glomerular endothelial cells in the recovering glomerulus with rosiglitazone treatment, although rosiglitazone did attenuate the clinical course of glomerulonephritis.

## 9. CONCLUSIONS AND PERSPECTIVES

Activation of the PPAR-*γ*-receptors present on renal endothelial cells affects their cellular behavior. Proliferation, apoptosis, migration, and angiogenesis by endothelial cells are modulated, but may involve both stimulation and inhibition depending on the specific circumstances. It remains unclear how this bimodal potential of PPAR-*γ*-agonists is regulated. An important consequence of PPAR-*γ*-receptor activation is the enhanced production of nitric oxide and C-type natriuretic peptide and superoxide dismutase, while endothelin-1 production is inhibited. Together, this improves the capacity of the endothelium to exert vasodilatory, anti-inflammatory, and antioxidative actions, resulting in blood pressure lowering and direct renoprotective effects. In addition, the presentation of adhesion molecules and release of cytokines aimed at recruiting inflammatory cells to activated endothelium is inhibited by PPAR-*γ*-agonism. This may also in part account for the anti-inflammatory effects of PPAR-*γ*-agonists, supplementary to direct effects on the inflammatory cells themselves. Finally, PPAR-*γ*-receptors are also found on endothelial progenitor cells and PPAR-*γ*-agonist stimulate progenitor-mediated endothelial repair, although definitive evidence that this occurs in the kidney is currently lacking.

Activation of other PPAR-receptors besides PPAR-*γ* may also have beneficial effects on the endothelium. Recently, selective agonists for PPAR-*β*/*δ* were developed [111]. PPAR-*β*/*δ* receptors are present on endothelium and stimulation with a pharmacological PPAR-*β*/*δ*-agonist was found to increase endothelial cell proliferation and angiogenesis in vitro and in vivo, mainly mediated through VEGF [112]. Like PPAR-*γ*-agonists, PPAR-*β*/*δ*-agonists increase circulating endothelial progenitor cell levels, augment endothelial progenitor cell function in vitro, and improve endothelial progenitor cell-mediated neovascularization of ischemic tissue [113]. Also, much like PPAR-*γ*-agonists, PPAR-*β*/*δ*-agonist treatment reduced the expression of adhesion molecules and monocyte binding to activated endothelial cells [114].

Currently, PPAR-*γ*-agonist treatment is not standard clinical practice in nondiabetic renal patients. As shown in this review, experimental studies indicate that stimulatory effects of PPAR-*γ*-agonism on endothelium may provide additional benefit in nondiabetic renal disease. Together with other potentially therapeutic effects independent of the insulin-sensitizing action such as the anti-inflammatory actions, this provides a rationale for further clinical evaluation in nondiabetic renal patients. For patients with diabetic kidney disease, a pressing question is whether glycemic control with a TZD is superior to other antidiabetic drugs for preventing the decline of renal function. To date, only retrospective studies, post hoc analyses, and pilot studies are available to help answer this question. Trials designed to specifically evaluate this question have yet to be performed.
